# Therapeutic Effects of the Combination of Alpha-Lipoic Acid (ALA) and Coenzyme Q10 (CoQ10) on Cisplatin-Induced Nephrotoxicity

**DOI:** 10.1155/2020/5369797

**Published:** 2020-04-09

**Authors:** Eman Aly Khalifa, Ahmed Nabil Ahmed, Khalid Shaaban Hashem, Ahmad Gad Allah

**Affiliations:** ^1^Department of Biotechnology and Life Sciences, Faculty of Postgraduate Studies for Advanced Sciences, Beni-Suef University, Beni-Suef, Egypt; ^2^International Center for Materials Nanoarchitectonics (WPI-MANA), National Institute for Materials Science (NIMS), Tsukuba, Japan; ^3^Department of Biochemistry, Faculty of Veterinary Medicine, Beni-Suef University, Beni-Suef, Egypt; ^4^Department of Physiology, Faculty of Medicine, Al Azhar University, Assiut, Egypt

## Abstract

**Background:**

Nephrotoxicity of cisplatin has been recognized since its introduction more than 25 years ago. However, despite intense efforts to develop less toxic and equally effective alternatives, cisplatin continues to be widely prescribed. *Aim and Objectives*. The study is aimed at assessing the possible prophylactic effect of coenzyme Q10 (CoQ10) and alpha-lipoic acid (ALA) (separately or in combination) on experimentally cisplatin-induced nephrotoxicity. *Subjects and Methods*. An experimental study was performed on adult male albino rats (*n* = 40), weighing 200–250 g. Rats were randomly divided into 5 groups: group I (normal saline control), group II (cisplatin control), group III (CoQ10 and cisplatin), group IV (ALA and cisplatin), and group V (CoQ10, ALA, and cisplatin). CoQ10 and/or ALA were given as pretreatment for 9 days, followed by cisplatin injection in the 10th day of the study, followed by a short posttreatment course for 3 days. Renal functions, tissue antioxidant activity, and inflammatory markers (tumor necrosis factor, TNF) were estimated along with histopathological study.

**Results:**

Renal function tests and urinary proteins were significantly higher within group II compared with other groups (*P* value <0.001). Creatinine clearance was significantly higher with combination therapy (group V compared to other groups). Both TNF and malondialdehyde (MDA) were significantly higher within group II whereas GSH content, catalase, and superoxide dismutase (SOD) were significantly lower in group II. MDA level was significantly lower when combination therapy was used. Marked renal damage was histologically detected in the cisplatin group, whereas the least renal damage was noticed in the combination group.

**Conclusion:**

The study confirmed the role of antioxidants in preventing nephrotoxicity caused by cisplatin; the prophylactic effect of combined therapy with CoQ10 and ALA is superior to that of monotherapy.

## 1. Introduction

Cisplatin (cis-diamminedichloroplatinum [II], CDDP) is the most significant platinum anticancer medication that is generally used to treat a variety of tumors including head, neck, and lung malignancies [1]. Reduction rates approached >90% in testicular malignancy [2]. However, significant side effects were reported by the administration of high doses of cisplatin. The major side effects include neurotoxicity, ototoxicity, and nephrotoxicity [3]. It was found that renal dysfunction occurs in more than 70% of pediatric patients treated with cisplatin [4].

Numerous processes and mechanisms lead to cisplatin nephrotoxicity and contribute to its complexity. However, the generation of toxic reactive oxygen species (ROS) remains a significant causative agent [3]. An important contributor for cisplatin nephrotoxicity is its take-up into kidney cells. The uptake of cisplatin by kidney cells is much higher than any tissues, especially in proximal tubules of the kidney where cisplatin concentration can reach up to five folds higher than that of the serum [5]. Cisplatin additionally represses the antioxidant enzymes including glutathione S-transferase, glutathione peroxidase, and superoxide dismutase, prompting lethal degrees of ROS inside the cell [6]. The resulting ROS suppresses the respiratory chain and ATP generation. This leads to disturbance of the function of cells and destruction of cell proteins, lipids, and nucleic acids; prompts endoplasmic reticulum stress; and causes cell necrosis [7]. Other mechanisms that may play a role in nephrotoxicity by cisplatin include inflammation [8] and cell apoptosis [9].

Several lines of evidence suggest that mitochondrial DNA or other mitochondrial targets are perhaps more important than nuclear DNA damage in mediating cisplatin-induced cell death. Cisplatin is hydrolyzed to generate a positively charged metabolite which preferentially accumulates within the negatively charged mitochondria. Thus, the sensitivity of cells to cisplatin appears to correlate with both the density of mitochondria and the mitochondrial membrane potential. This observation may explain the particular sensitivity of the renal proximal tubule to cisplatin toxicity, as this segment exhibits one of the highest densities of mitochondria in the kidney [10].

An antioxidant is defined as any substance that, when present at low concentrations, delays or prevents oxidations of cell components like lipids, proteins, carbohydrates, and DNA. Superoxide dismutase (SOD), catalase (CAT), and glutathione reductase represent the first line antioxidants within the human body [11].

There are two important metabolic oxidants in the mitochondria, namely, coenzyme Q (CoQ10) and lipoic acid. The presence of these molecules shows that mitochondria as individual organisms can defend themselves against the harmful effects of the oxygen atmosphere [12].

Alpha-lipoic acid (ALA) is derived from octanoic acid. In the mitochondria, it acts as a cofactor of mitochondrial *α*-ketoacid dehydrogenase [13]. ALA increases the level of antioxidant enzymes and aids in recycling of vitamins E and C. Because of these properties, ALA is known as the “universal antioxidant.” Besides, ALA has anti-inflammatory action apart from its antioxidant activity. The anti-inflammatory effects of LA are due to suppression of inflammatory activity of NF-kB, TNF-a, and IL-6 and increased anti-inflammatory proteins, such as nuclear erythroid 2-related factor (Nrf2) [14].

In vivo lipoic acid is easily converted into its reduced form, dihydrolipoic acid (DHLA). For a long time, ALA has been known as a cofactor of a-ketoacid dehydrogenases, but recent research has been focused mainly on the antioxidative properties of ALA and DHLA. These two compounds have been reported as effective free radical scavengers and metal chelators acting in vitro as well as in vivo. DHLA can regenerate reduced forms of essential intracellular antioxidants such as ascorbate and tocopherol. Moreover, ALA has been reported to affect the activities of transcription factors, NF-kB and AP-1 [15].

Furthermore, ALA has the ability to chelate toxic metals in a direct way or through its ability to increase glutathione levels inside the cells. Because of these significant antioxidant activities, ALA is used to protect against oxidative damage in various diseases such as diabetes, neuropathy, cataracts, and aging [16–18].

Coenzyme Q10 (CoQ10) is a lipid-soluble vitamin-like substance, also known as ubiquinone. CoQ10 is similar to vitamin K in chemical composition; however, it is not considered as a vitamin because it can be synthesized inside the body. CoQ10 is found in the membranes of cell organelles, especially the inner mitochondrial membrane. It is the single naturally occurring lipid soluble antioxidant that can be synthesized endogenously. It participates in aerobic cellular respiration working as an electron carrier in the process creating energy through the formation of ATP (adenosine triphosphate) [19–21]. CoQ10 has been demonstrated to be a regulator of vasoactive reactions; thus it was extensively studied in prevention and treatment of diabetes, cardiovascular, and renal disease [12].

For several years, coenzyme Q (CoQ10 in humans) was known for its key role in mitochondrial bioenergetics; later studies demonstrated its presence in other subcellular fractions and plasma and extensively investigated its antioxidant role. These constitute the basis on which research supporting the clinical use of CoQ10 is founded. Also, at the inner mitochondrial membrane level, coenzyme Q is recognized as an obligatory cofactor for the function of uncoupling proteins and a modulator of the transition pore [19].

In the reduced form as ubiquinol, CoQ10 inhibits the peroxidation of cell membrane lipids and can act as antioxidant outside the mitochondrial membrane. Not only can it recycle and regenerate other antioxidants such as vitamins E and C, but it can also uniquely affect the initiation and propagation of ROS [22].

Another notable function of CoQ10 would be the ability to interact with dihydrolipoic acid (DHLA), by transferring a pair of electrons. This helps to keep CoQ10 in the reduced state, thereby maximizing antioxidant capacity in other extramitochondrial membranes [23]. The modulatory effect of CoQ10 on gene expression has also been reported [24].

It was found that antioxidants either work separately or collaborate when they are oxidized [16]. The generation of free radicals certainly plays a central role in the development of different pathological conditions. Most of these pathological conditions are multifactorial in origin. Hence, the use of a combination of antioxidants, rather than antioxidant monotherapy, is strongly recommended [12].

**Figure 1:**
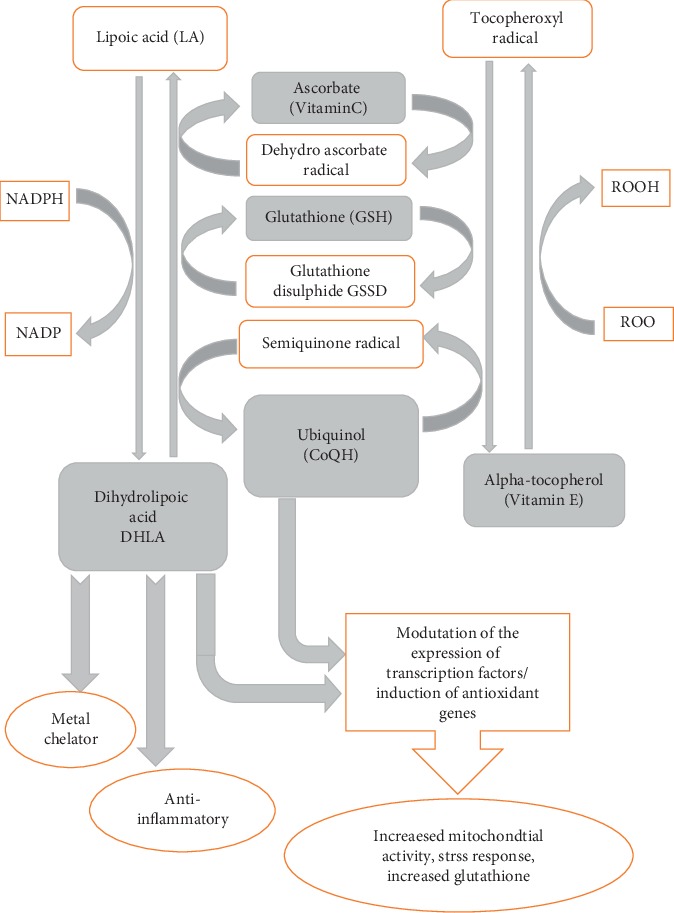
Interactions between CoQ10, ALA, and other antioxidants.

The abovementioned data suggests that ALA and CoQ10 share the following common properties that may facilitate their synergistic activity. First, CoQ10 can interact with DHLA to remain in the reduced state. Second, both work as direct antioxidants and ROS scavengers. Third, both increase the activity of glutathione and other antioxidant enzymes. Fourth, both contribute to the recycling of vitamins E and C. Last, ALA and CoQ10 modulate the transcription factors and gene expression responsible for mitochondrial activity and stress response (Figure 1). For these reasons, the authors hypothesized that this combination may be beneficial in reducing cisplatin nephrotoxicity. The study was planned to investigate the possible prophylactic benefits of combining CoQ10 and ALA as an early treatment against cisplatin-induced nephrotoxicity.

## 2. Subjects and Methods

### 2.1. Technical Design

The present study has been completed in biochemistry labs at Beni-Suef University, Egypt.

### 2.2. Inclusion Criteria

This study has been performed on adult male albino rats (Wistar strain) (*n* = 40), weighing 200–250 g. Rats, 8–10 weeks of age, were housed in a controlled situation with a 12 h light/dark cycle, at 23 ± 2°C, 50 ± 5% relative humidity. Precautions were implemented to avoid exposure of rats to any type of stress.

### 2.3. Animal Groups

Rats had been permitted to adapt for about fourteen days before the experimentation, and afterward they were haphazardly separated into five groups and permitted to have free access to an ordinary laboratory rat diet and tap water. Each group contained 8 rodents; groups were classified as follows:

  Group I (normal saline group): normal saline 0.5 ml was given by intraperitoneal infusion (IP) on the tenth day of the trial.
  Group II (cisplatin control): cisplatin (6 mg/kg) in isotonic saline was given by intraperitoneal infusion (IP) on the tenth day of the trial.
  Group III (coenzyme Q 10 group): a daily portion of CoQ10 (100 mg/kg) was given orally for 13 days, and cisplatin (6 mg/kg) was given (IP) on the tenth day of the test.
  Group IV (lipoic acid group): ALA (100 mg/kg) was given orally for 13 days, and cisplatin 6 mg/kg (IP) on the tenth day of the test.
  Group V (combination group): this group was given both ALA (100 mg/kg) daily and CoQ10 (100 mg/kg) orally for 13 days. Cisplatin (6 mg/kg) was given (IP) on the tenth day of the test.

### 2.4. Operational Design

Doses and timing were chosen based on other related researches [25–27]. Animals had been sacrificed 72 hrs after cisplatin or saline injection, and then blood and kidney tissue samples were collected.

#### 2.4.1. Chemicals and Drugs

Cisplatin vials (Bristol Myers Squibb Co., USA) were utilized. DL-*α*-lipoic acid was purchased from Sigma-Aldrich Chemie, Germany. CoQ10 (100) mg was purchased from California Gold Nutrition®, USA. Every single other compound and reagents utilized were of the most noteworthy virtue grade accessible. CoQ10 suspension was prepared in 0.9% of normal saline and administered orally to group III (alone) and group v (combined with ALA) for 13 days. Lipoic acid group (group IV) was given a daily dose of lipoic acid (100 mg/kg) orally for 13 days; it was also given to group V (the combination group).

#### 2.4.2. Animals

Adult male Wistar albino rats weighing 200–250 g were purchased from the Egyptian Organization for Biological Products and Vaccines (VACSERA, Giza, Egypt).

### 2.5. Preparation of the Kidneys

Both kidneys were rapidly excised and weighed. The renal cortex was then separated and kept at −8°C. Then it was homogenized using cold potassium phosphate buffer (0.05 M, pH 7.4), followed by centrifugation for obtaining the supernatant. Kidneys were then cut longitudinally, followed by fixation in 10% formalin, and then they were embedded in paraffin for histopathological examination.

### 2.6. Renal Homogenate

Renal homogenate was utilized for assessment of total protein level and antioxidant parameters within the kidney; these include renal tissue glutathione (GSH), superoxide dismutase (SOD), and catalase activities.

#### 2.6.1. Measurement of Tissue Glutathione (GSH) Content

Reduced GSH was estimated spectrophotometrically at 412 nm as indicated by the technique for Ellman [28]. T-GSH assay kit E-BC-K097-M, Elabscience®, USA, was used. To each microcentrifuge tube containing 0.5 ml TCA-EDTA, 0.5 ml of diluted samples was added. The tubes were gently shaken intermittently for 15 minutes, followed by centrifugation at 2000 rpm at room temperature for 5 minutes. An aliquot 0.1 of the resultant clear supernatant was aspirated and mixed with 1.7 ml phosphate buffer in separate test tubes. A duplicate was made for each sample, and then 0.1 ml Ellman's reagent was added to each tube. After 5 min, the optical density was measured at 412 nm against a blank using a spectrophotometer. A set of serial dilutions of reduced GSH were used to construct a calibration curve. The measure of GSH was expressed as *μ*mol/g tissue.

#### 2.6.2. Measurement of Superoxide Dismutase (SOD) Activity

SOD activity was determined depending on the inhibition of pyrogallol oxidation by SOD [29]. Pyrogallol solution was obtained from Sigma-Aldrich (product number 254002). The other chemicals used in the test were purchased locally. An aliquot of diluted sample (100 *μ*l) was added to 25 *μ*l of pyrogallol (24 mmol/L prepared in HCl), and the final volume was adjusted to 3 ml using Tris HCl (0.1 M, pH 7.8). The changes in the absorbance at 420 nm were recorded for 3 minutes at 1 min interval, using a spectrophotometer. One unit of SOD activity is defined as the amount of the enzyme causing 50% inhibition of autooxidation of pyrogallol. SOD activity was expressed as U/mg protein (or units/min/g wet tissue).

#### 2.6.3. Measurement of Catalase Activity

This was estimated depending on the decrease in absorbance at 240 nm due to decomposition of hydrogen peroxide by catalase [30]. Catalase (CAT) assay kit E-BC-K031-S, Elabscience®, USA, was used. In a 3 ml quartz cuvette, 100 *μ*l of homogenate (10%) was added to 2.9 ml of 19 mmol/L H_2_O_2_ solution prepared in potassium phosphate buffer (0.1 M, pH 7.4). The reaction was monitored by continuous recording of the change in the absorbance at 240 nm every minute for 2 min using Jenway 6305 UV/visible spectrophotometer, expressed as U/g wet tissue.

### 2.7. Assessment of Renal Lipid Peroxidation

This was evaluated by measuring malondialdehyde (MDA) in tissues which is broadly acknowledged as a marker of lipid peroxidation. MDA is formed from the breakdown of polyunsaturated fatty acids and serves as a convenient index for determining the extent of the peroxidation reaction. MDA was measured based on colourimetric determination of thiobarbituric acid reactive substance (TBARS) [31]. MDA assay kit (ab118970) was purchased from Abcam distributed by GeneTECH (Egypt) (TBARS). Determination of MDA is based on the reaction of one molecule of MDA with two molecules of thiobarbituric acid at low pH (2-3). An aliquot of 0.5 ml of diluted sample was pipetted into a 10 ml centrifuge tube, followed by the addition of 3 ml of 1% orthophosphoric acid and 1 ml of 0.6% thiobarbituric acid. The tubes were incubated in a water bath at 95°C for 45 min. After cooling the mixture, 4 ml of n-butanol was added to each tube and mixed vigorously. The butanol phase (upper layer) was separated by centrifugation at 2000 rpm for 10 min. The color was measured at 535 and 520 nm using a spectrophotometer. The difference in optical density between both wave lengths was used as a measure of MDA absorbance. A calibration curve was constructed using serial dilutions of 1,1′,3,3′-tetramethoxypropane. The results were expressed as nmol/g tissue.

### 2.8. Quantitative Analysis of Tumor Necrosis Factor-*α* (TNF-*α*)

This was done by ELISA quantitative enzyme immunoassay technique, Quantikine® ELISA, USA (TNF-*α* is a cisplatin-induced inflammatory cytokine). A monoclonal antibody specific for rat TNF-*α* was precoated onto a microplate. Standards, control, and samples were pipetted into the wells, and any TNF-*α* present was bound by the immobilized antibody. After washing away any unbound substances, an enzyme-linked polyclonal antibody specific for rat TNF-*α* was added to the wells. Following a wash to remove any unbound antibody-enzyme reagent, a substrate solution was added to the wells. The enzyme reaction yields a blue product that turns yellow when the stop solution was added. The intensity of the color measured was in proportion to the amount of TNF-*α* bound in the initial step. The sample values were then read off from the standard curve.

### 2.9. Histopathological Examination

Kidney tissues were kept in 10% formalin, and dehydration was done by alcohols, followed by clearance by xylene and then insertion in paraffin wax. Sections were then stained using hematoxylin and eosin. The amplification intensity of 400 was utilized to demonstrate the histopathological changes utilizing light microscopy.

### 2.10. Biochemical Examination of Blood and Urine Samples

  Blood samples were used to measure serum creatinine, blood urea, and serum uric acid. Reagents were obtained from BIOMED diagnostics, Egypt: urea (URE118100), uric acid (UA119090), and creatinine (CRE 106100).
  Urine samples were utilized for determination of urinary albumin and total protein. The urinary creatinine was estimated to measure creatinine clearance. Reagents were obtained from Diamond Diagnostics, USA, distributed by Lab Supply, Egypt: albumin (BK-467858D) and total protein (BK-465986D).

### 2.11. Protocol Approval by the Ethical Committee

Before the establishment of the study, based on local regulation, the study protocol was submitted and approved by the Ethical and Research Committee (Beni-Suef University Institutional Review Broad (IRB)).

### 2.12. Data Management and Statistical Analysis

Data processing and statistical analysis were done using MedCalc ver. 18.2.1 (MedCalc, Ostend, Belgium). Kruskal–Wallis one-way ANOVA test was the main test used in the study. It was used to compare nominal variables among three or more groups. Other less frequently used tests included Wilcoxon's test that was used to compare the control group with any of the other groups; Chi-square and logistic regression analysis were used for categorical variables. Spearman's correlation was used for continuous and ordinal variables. The last three tests were used mainly in an attempt to relate the histopathological findings to the treatment groups. *P* values less than 0.05% were considered to be statistically significant, and less than 0.001 highly significant.

## 3. Results

### 3.1. Biochemistry Results

**Figure 2:**
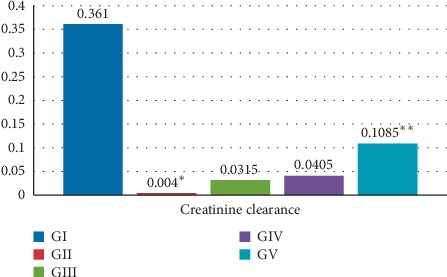
Creatinine clearance in the studied groups. Creatinine clearance was significantly reduced when group II was compared to other groups. ^*∗*^*P* value was 0.00081. Combination therapy resulted in a significant elevation of creatinine clearance when compared to monotherapy (i.e., group V compared to group III and group IV). ^*∗∗*^*P* value was 0.0176.

One mortality was reported in group II (48 hours after cisplatin injection), with no reported mortality among other groups. Blood urea, serum creatinine, serum uric acid, urinary albumin, and total protein levels were significantly higher within group II compared with group V, group IV, and group III (*P* value <0.001); these are shown in Table 1. No significant difference was found among the groups treated with ALA and/or CoQ10 regarding these parameters (groups III, IV, and V). Similarly, it was found that creatinine clearance was significantly reduced when group II was compared to other groups. When combination therapy was compared to monotherapy (group V compared to group III and group IV), it was found that combination therapy resulted in a significant elevation of creatinine clearance (*P* value <0.05), Figure 2.

### 3.2. Oxidative Stress Markers and Inflammatory Markers

**Figure 3:**
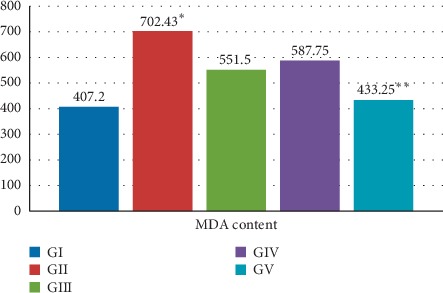
MDA content in the studied groups. MDA was significantly higher when group II was compared to other groups. ^*∗*^*P* value was 0.026. Combination therapy resulted in a significant reduction of MDA when compared to monotherapy (i.e., group V compared to group III and group IV). ^*∗∗*^*P* value was 0.014.

TNF (an inflammatory cytokine) and MDA levels were significantly higher within group II compared to groups III, IV, and V. Meanwhile GSH content, catalase, and SOD were significantly lower within group II compared to other groups (*P* value <0.05). Treatment with CoQ10 and/or ALA resulted in a significant reduction in the MDA level caused by cisplatin. Moreover, when group V (combined therapy) was compared to monotherapy groups (groups III, IV), it was found that combined therapy is more effective in reducing MDA level than monotherapy; this difference was statistically significant (*P* value <0.05) (Table 2, Figure 3).

### 3.3. Histopathological Findings

**Figure 4:**
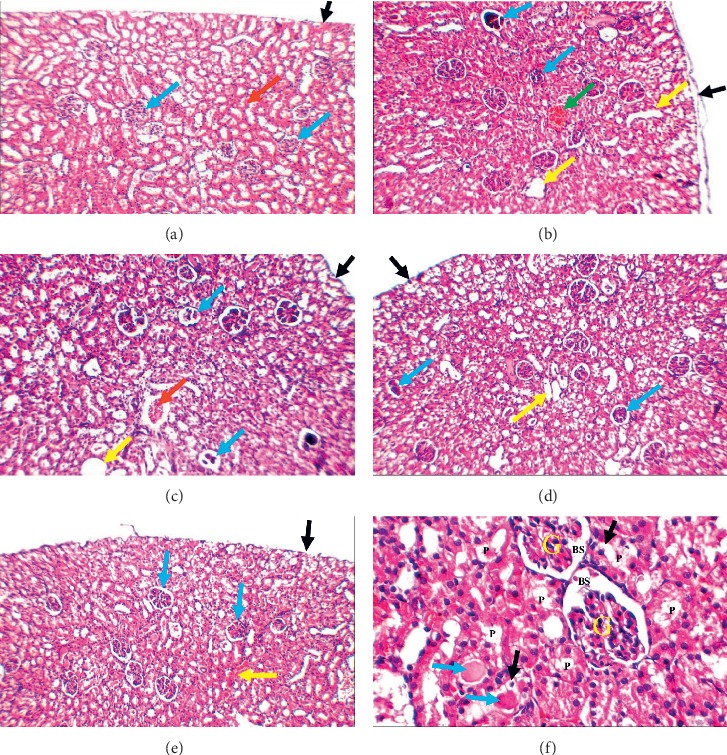
(a) Control: kidney showing average renal capsule (black arrow), average glomeruli (blue arrows), average tubules (red arrow), and average interstitium (H&E ×200). (b) Cisplatin: kidney showing partially detached renal capsule (black arrow), scattered atrophic glomeruli (blue arrows), dilated tubules (yellow arrows), and markedly dilated congested interstitial blood vessels (green arrow) (H&E ×200). (c) Cisplatin + CoQ10: kidney showing average renal capsule (black arrow), scattered atrophic glomeruli (blue arrows), dilated tubules (yellow arrows), and dilated congested blood vessel (red arrow) (H&E ×200). (d) Cisplatin + ALA: kidney showing average renal capsule (black arrow), scattered small-sized glomeruli (blue arrows), and dilated tubules (yellow arrow) (H&E ×200). (e) Cisplatin + CoQ10 + ALA: kidney showing average renal capsule (black arrow), average glomeruli (blue arrows), and mildly congested interstitial blood vessels (yellow arrow) (H&E + 200). (f) Cisplatin + CoQ10 + ALA: high power view showing average glomeruli (G) with average Bowman's spaces (BS), proximal tubules (P) with edematous epithelial lining (black arrows) and preserved brush borders, and intratubular hyaline casts (blue arrows) (H&E ×400).

A modified pathological scoring system was used to subjectively assess 8 morphological parameters; the given scores ranged from 0 to ++ [32]. It was noted that marked renal damage occurred in cisplatin treated group (group II), and the least renal damage was noticed in the combination group (group V) (Figure 4). A detailed description was as follows;

  Group I (control): kidney showed average renal capsule, average glomeruli with average Bowman's spaces, average proximal tubules with preserved brush borders, and average distal tubules, and renal medulla showed average collecting tubules with average interstitium.
  Group II (cisplatin): kidney showed partially detached renal capsule, scattered atrophic glomeruli with widened Bowman's spaces, proximal tubules with edematous, apoptotic and necrotic epithelial lining, complete loss of brush borders, intratubular hyaline casts, and markedly dilated congested interstitial blood vessels, and renal medulla showed markedly dilated collecting tubules, with atrophied epithelial lining, and intratubular hyaline casts.
  Group III (cisplatin + CoQ10): kidney showed average renal capsule, scattered atrophic glomeruli with widened Bowman's spaces, proximal tubules with edematous and apoptotic epithelial lining and partial loss of brush borders, intratubular cellular debris and hyaline casts, and markedly dilated congested interstitial blood vessels with areas of hemorrhage, and renal medulla showed collecting tubules with edematous and apoptotic epithelial lining, mildly dilated congested blood vessels, and intratubular hyaline casts.
  Group IV (cisplatin + ALA): kidney showed average renal capsule, scattered small-sized glomeruli with widened Bowman's spaces, proximal tubules with edematous and apoptotic epithelial lining, partial loss of brush borders, and mildly dilated congested interstitial blood vessels, and renal medulla showed collecting tubules with average epithelial lining and intratubular hyaline casts.
  Group V (cisplatin + CoQ10 + ALA): Kidney showed average renal capsule, average glomeruli with average Bowman's spaces, proximal tubules with edematous and apoptotic epithelial lining, partial loss of brush borders, intratubular hyaline casts, and mildly dilated congested interstitial blood vessels, and renal medulla showed collecting tubules with edematous epithelial lining and intratubular cellular debris.  Table 3 summarizes the histopathological findings in the different studied groups.

## 4. Discussion

Despite the fact that different compounds may be termed ‘‘antioxidants,” still vitamin E, vitamin C, coenzyme Q10 (CoQ10), and alpha-lipoic acid (ALA) are among the most extensively studied ones. Cumulative data have proved that ALA exhibits significant protective antioxidant effects against oxidative damage encountered in several diseases such as diabetes mellitus, neuropathy, renal diseases, and aging [16–18]. CoQ10 has been also shown to be a powerful antioxidant. Its role in the prevention and treatment of diabetes, cardiovascular, and renal diseases was investigated in different studies [19].

The protective role of CoQ10 when combined with ALA in prevention of nephrotoxicity was not fully investigated. This study was an attempt to evaluate the potential synergistic activity of CoQ10 and ALA during cisplatin-induced nephrotoxicity.

All antioxidants work in a coordinated system to control levels of free radical formation, and deficiencies in one element can affect the others. As antioxidants, CoQ10 and ALA are interrelated, complementary to each other, and involved in the different pathways that generate and recycle other antioxidants, mainly glutathione, vitamin E, and vitamin C (Figure 1). Glutathione, an important water-soluble antioxidant, directly quenches ROS such as lipid peroxides and also plays a major role in the body's detoxification process. Research suggests that glutathione and vitamin C work interactively to quench free radicals and that they have a sparing effect upon each other [33].

In extracellular fluids, vitamin C is considered the most important water-soluble antioxidant. It is capable of neutralizing ROS in the aqueous phase before lipid peroxidation is initiated. Vitamin E, a major lipid-soluble antioxidant, is the most effective chain-breaking antioxidant within the cell membrane where it protects membrane fatty acids from lipid peroxidation. Vitamin C has been reported as being capable of regenerating vitamin E. [34]. As vitamin E also plays a role in free radical attainment, CoQH_2_ can regenerate vitamin E from subsequent *α*-tocopherol radical formation. In animal models, depleted levels of *α*-tocopherol were followed by the subsequent oxidation of ubiquinol. This suggests that *α*-tocopherol and ubiquinol act in concert to quench free radicals [35].

The synergistic interaction between LA and Q10 was demonstrated by Wagner et al. in cultured skeletal muscle cells; they found that this combination significantly increased nuclear levels of PGC1*α*, a master switch of energy metabolism and mitochondrial biogenesis. Furthermore, supplementation with LA plus Q10 resulted in an increase of genes encoding proteins involved in stress response, GSH synthesis, and its recycling. In ALA-plus-Q10-treated muscle cells, a significant 4-fold increase in GSH was evident. This increase in GSH was accompanied by increased nuclear Nrf2 (nuclear factor erythroid-like 2 derived factor 2) protein levels, partly regulating *γ*GCS (*γ*-glutamylcysteine-synthetase) and GST (glutathione-S-transferases) gene expression. These led to the improvement of energy homeostasis, stress response, and antioxidant defense mechanisms. Treatment with combined therapy resulted in the elevation of glutathione levels to higher levels compared to monotherapy [36].

In our study, the difference among groups treated with monotherapy versus combined therapy was not significant in all aspects; however, there are two significant findings in favor of combined therapy. The first is the improved creatinine clearance in the group treated with combined treatment (creatinine clearance is an important marker for renal function). The second is the significant decrease in the MDA level among the combination group (MDA is an important marker for lipid peroxidation); this reflects the effectiveness of the combined therapy in reducing the oxidative stress mediated by cisplatin. A third notable finding that supports combined therapy is the less kidney damage detected by histopathology when compared with each of the monotherapy groups.

Lipid peroxidation involves the loss of hydrogen from a polyunsaturated fatty acid, resulting in a peroxyl radical. Reduced CoQH_2_ (ubiquinol) loosely holds electrons and acts to eliminate lipid peroxyl radicals by either directly producing semiquinone radical (CoQH·) or scavenging other peroxyl radicals [36]. In our study, there was a significant reduction of MDA level when CoQ10 or ALA was used. Moreover, the combined therapy was more significant than monotherapy in reducing MDA and hence the oxidative stress. Similar results were given by Ahmadvand et al. while examining the possible protective effect of CoQ10 in vivo and in vitro regarding lipid peroxidation, antioxidant enzyme activity, and glomerulosclerosis in alloxan-induced type 1 diabetic rats. They found that coenzyme Q10 significantly antagonizes the elevated levels of MDA in serum and kidney in the treated group compared with the diabetic untreated group. They concluded that CoQ10 exerts beneficial effects on the lipid peroxidation and antioxidant enzyme activity in alloxan-induced type 1 diabetic rats [37].

The effect of monotherapy with CoQ10 in diminishing renal injury mediated by cisplatin was investigated by Fouad et al. In that study CoQ10 treatment was started one day before cisplatin injection and continued for 6 consecutive days. CoQ10 caused a significant reduction in blood urea nitrogen and serum creatinine levels, which were increased by cisplatin. CoQ10 significantly compensated deficits in the antioxidant defense mechanisms (reduced GSH level and SOD activity), suppressed lipid peroxidation, and decreased the elevations of TNF-*α*. Also, histopathological renal tissue damage mediated by cisplatin was ameliorated by coenzyme Q10 treatment [38]. The results of that study are comparable to the results of our study even though CoQ10 was given for 6 days after cisplatin injection in the other study and was given for 3 days only in our study. This may support the idea that giving a pretreatment course (9 days in our study) is beneficial and comparable with the posttreatment course.

The role of ALA in preventing kidney injury was confirmed in different studies. This led us to combine ALA with CoQ10 in the current study to maximize the potential therapeutic benefits. An example of these studies is the one conducted by Pianta et al. They demonstrated that ALA plays an important role in the amelioration of cisplatin-induced acute kidney injury. They found that ALA treatment attenuated renal tubular injury scores (*P* < 0.05) and decreased serum creatinine and urinary damage biomarkers of proximal tubular injury. Protection was demonstrated by reduced structural damage, improved glomerular filtration, and reduced excretion of urinary biomarkers of proximal tubular damage [39]. These data are comparable to our data in the groups treated with ALA; however, the renal damage in our study may be objectively higher since ALA was given mainly as prophylactic therapy and continued only for 3 days after cisplatin exposure; on the other hand, the histological findings were almost comparable when the combination treatment was given.

Another study by Jamor et al. investigated the impact of ALA against kidney injury in alloxan-induced diabetic rats. ALA (100 mg/kg) was given intraperitoneally (IP), daily to 6 weeks. Treatment with ALA led to a significant elevation in catalase and GSH levels with a reduction in MDA levels. Histopathological lesions such as increased glomerular volume and lymphocyte infiltration were attenuated in the ALA treated group. They concluded that ALA supplementation is effective in preventing complications induced by oxidative stress and atherosclerosis in diabetic rats [40]. In contrast to our study, ALA was given for 6 weeks and this resulted in a better histopathological outcome.

It is worth mentioning that ALA has different modes of action that contribute better to the alleviation of the toxic damage in the kidney caused by cisplatin or other compounds having heavy metals in their structure. For example, the protective effect of ALA has been tested against cadmium-induced nephrotoxicity by Luo et al. Their findings indicated that the antioxidant, cadmium chelation, and antiapoptotic activities of ALA are the key factors that alleviate nephrotoxicity [41]. De Sousa Alegre et al. reported that ALA alone increases urinary osmolality, decreases urinary urea and creatinine, and diminishes urinary content of uric acid [17].

Our study design was focused on the prophylactic effect of both ALA and CoQ10, rather than treatment of kidney injury; thus, the treatment was given 9 days before the injection of cisplatin, and the samples were taken 72 hours after cisplatin administration. In most of the abovementioned studies, concomitant administration of cisplatin and the tested agent were applied; however, our data are in general comparable to these studies. Adding ALA to CoQ10 in the current study improved the outcome of our proposed prophylactic course. This raises the idea that giving a pretreatment course along with posttreatment course may result in possibly better outcome; this point needs further study and investigation.

## 5. Conclusion

The current study demonstrated the beneficial cumulative effect of combining CoQ10 and ALA in reducing the toxic damage caused by cisplatin to the kidney. More studies are needed to compare the results of this pretreatment combined regimen with a posttreatment regimen.

## Figures and Tables

**Table 1:** Biochemistry data in the studied groups.

	Group I (*N* = 8)	Group II (*N* = 7)	Group III (*N* = 8)	Group IV (*N* = 8)	Group V (*N* = 8)	*P* value in group II (cisplatin) versus other groups	*P* value in group V (combination) versusgroups III, IV (monotherapy)
Urea (mg/dl) Mean ± SD	17.36 ± 4.61	227.7 ± 9.3	171.13 ± 6.19	162.8 ± 5.99	158.38 ± 6.86	0.00081^*∗*^	NS

Urinary albumin (mg/24 h) Mean ± SD	23.61 ± 11.3	478.6 ± 37.6	257.8 ± 12.1	289.13 ± 17.2	231 ± 4.59	0.00093^*∗*^	NS

Urinary total protein (mg/24 h) Mean ± SD	302.32 ± 62.9	3113.8 ± 118.4	1503.3 ± 69.4	1495.9 ± 106.4	1296.8 ± 75.4	0.00086^*∗*^	NS

Uric acid (mg/dl) Mean ± SD	3.7 ± 0.246	8.33 ± 0.164	3.92 ±0.0797	3.99 ± 0.111	3.69 ± 0.066	0.00075^*∗*^	NS

Creatinine (mg/dl) Mean ± SD	1.5 ± 0.541	6.01 ± 0.376	3.38 ± 0.227	3.801 ± 0.178	2.96 ± 0.097	0.00065^*∗*^	NS

Creatinine clearance (ml/min) Median(range)	0.361 0.09–0.83	0.004 0.002–0.006	0.0315 0.028–0.038	0.0405 0.022–0.054	0.1085 0.096–0.199	0.00081^*∗*^	0.0176^*∗*^

^*∗*^Significant; NS: nonsignificant.

**Table 2:** Oxidative stress in the studied groups.

	Group I (N = 8)	Group II (N = 7)	Group III (N = 8)	Group IV (N = 8)	Group V (N = 8)	*P* value in group II (cisplatin) versus other groups	*P* value in group V (combination) versus groups III, IV (monotherapy)
TNF-*α*(pg/ml) Mean ± SD	29.45 ± 457	53.92 ± 0.477	47.22 ± 0.376	47.86 ± 0.162	46.02 ± 0.432	0.018^*∗*^	NS

MDA content (nmol/g wet tissue) Mean ± SD	407.21 ± 13.1	702.43 ± 33.21	551.5 ± 9.15	587.75 ± 13.4	433.25 ± 9.62	0.026^*∗*^	0.014^*∗*^

GSH content (mmol/g wet tissue) Mean ± SD	0.366 ± 0.0024	0.1726 ± 0.0059	0.2179 ± 0.0081	0.2011 ± 0.0072	0.2377 ± 0.0049	0.0167^*∗*^	NS

SOD (U/g wet tissue) Mean ± SD	7.57 ± 0.1077	4.16 ± 0.211	6.41 ± 0.1016	6.68 ± 0.055	6.18 ± 0.0529	0.009^*∗*^	NS

CAT (U/g wet tissue) Mean ± SD	0.595 ± 0.0041	0.4653 ± 0.0199	0.5244 ± 0.0063	0.5343 ± 0.0057	0.5728 ± 0.0079	0.011^*∗*^	NS

TNF: tumor necrosis factor. SOD: superoxide dismutase. MDA: malondialdehyde. GSH: glutathione content of renal tissue. CAT: catalase enzyme. ^*∗*^Significant. NS: nonsignificant.

**Table 3:** Histopathological results.

	Renal capsule	Glomeruli	BS	Tubular lining	Tubular lumen	Brush border	Interstitium	Medulla
Group I	0	0	0	0	0	0	0	0
Group II	+	++	+	++	++	++	++	++
Group III	0	++	+	+	++	+	++	+
Group IV	0	+	+	+	++	+	+	+
Group V	0	0	0	+	++	+	+	+

Renal capsule: 0: average +: partially detached ++: completely detached. Glomeruli (G): 0: average +: small-sized/congested ++: atrophied. Bowman's spaces (BS): 0: average + : widened/dilated ++: obliterated. Tubular lining: 0: average +: edematous/apoptotic ++: necrotic. Tubular lumen: 0: free +: cellular debris ++: hyaline casts. Brush border: 0: preserved +: partial loss ++: complete loss. Interstitium: 0: average +: mildly dilated BV ++: markedly dilated BV. Medulla: 0: average +: cellular debris/ hyaline casts ++: atrophied lining.

## Data Availability

The original data used to support the findings of this study are available from the corresponding author upon request.
